# Time Required to Create a Referral in Various Store-and-Forward Telemedicine Networks

**DOI:** 10.3389/fpubh.2019.00260

**Published:** 2019-09-18

**Authors:** Richard Wootton, Barry O'Kane

**Affiliations:** ^1^Norwegian Centre for Integrated Care and Telemedicine, University Hospital of North Norway, Tromsø, Norway; ^2^EndZone.IO Ltd., Glasgow, United Kingdom

**Keywords:** teleconsulting, low resource setting, referral preparation, user interface, store and forward

## Abstract

Store and forward telemedicine is used routinely in health care, but there is little published information about *how* such telemedicine systems are used. For example, an important aspect of the system's usability is the length of time it takes to submit a referral. Referral-submission times were measured in networks based on the Collegium Telemedicus system. In a 25-week period in 2018/2019, eight Collegium networks received a total of 1,649 clinical or educational cases submitted via the web interface. The time to prepare a referral was measured in 669 of these cases, in two different ways. An *indirect* measurement of the referral-preparation time was calculated as the interval between the user logging in, and the referral being submitted. A *direct* measurement of the referral-preparation time was calculated as the interval between the user opening the referral page and the referral being submitted to the server. The difference between the two measurements represents time spent by the user on other activities after logging in, before beginning the referral. The median referral-preparation time, measured directly, was 888 s (IQR 512-1765). The median of the differences between the two preparation times was 27s (IQR 8-146). The referral-preparation times in the eight networks were broadly similar, despite the differences in the nature of their operation (clinical or educational), and the types of case handled (single specialty or multi-specialty). Quantitative information about aspects of the user interface, such as the referral-preparation time, is important not only in the initial system design, but also in its subsequent development.

## Introduction

Store and forward telemedicine has been used routinely in health care for several decades. Yet there is little published information about *how* such telemedicine systems are used. For example, an important aspect of the system's usability is the length of time it takes to submit a referral: if referrals take too long to prepare, users will find other ways of answering their clinical questions (e.g., ordinary email) even though these are likely to be inferior to the use of store and forward telemedicine (1, 2).

Designers of telemedicine systems will usually have intuitive ideas about referral preparation and other matters, but these ideas are not likely to be founded on fact. Quantitative information about these aspects of the user interface are important not only in the initial system design, but also in its subsequent development.

Collegium Telemedicus offers a store-and-forward telemedicine system to support organizations conducting humanitarian or non-commercial work in low resource settings (3, 4). The objective of the present study was to measure the time users were taking to prepare cases for submission, with the aim of informing the future development of the system.

## Methods

Referral-preparation times were examined in the Collegium Telemedicus system, which at the time of the study comprised 33 clinical networks and 6 educational networks. A clinical case corresponded to a patient, and an educational case corresponded to a case report or a case-management discussion (5). The epoch examined was 8 September 2018 to 1 March 2019, a total of 25 weeks. Cases submitted via the Collegium mobile app were excluded, i.e., only cases submitted via the web interface were included in the present study.

The time taken to prepare a referral was calculated in two different ways:

An *indirect* measurement of the referral-preparation time was calculated as the interval between the user logging in, and the referral being submitted to the server; if subsequent referrals were submitted in the same session, they were ignored.
A *direct* measurement of the referral-preparation time was calculated as the interval between the user opening the referral page and the referral being submitted to the server.

Complex scenarios (see Appendix) during which a user allowed the session to time out, for example, were omitted. Thus, the present analysis was restricted to the simple situation where the user logged in, and after carrying out other operations such as reading messages concerning other cases, prepared the referral and submitted it. Note that whether a referral is to be transmitted via a store-and-forward telemedicine system, by plain email, or by instant messaging, the sender will probably need to summarize information from the patient record, obtain copies of clinical images and so forth, prior to initiating the process of electronic referral. Any prior (offline) time of this nature is not the subject of the present work.

A random sample of 1% of referrals were checked manually to confirm the classification into Simple or Complex.

The total size of any files uploaded with each referral was calculated in kByte.

## Results

During the study period, a total of 2,161 clinical cases were submitted. After excluding cases submitted via the Collegium mobile app, there were 1,649 cases which had been submitted via eight different Collegium networks (Table 1). Of these, 669 were classified as Simple cases and the referral-preparation times were calculated by both methods.

Indirect measurement. The median referral-preparation time was 1074 s (IQR 584-2035).
Direct measurement. The median referral-preparation time was 888 s (IQR 512-1765).

**Table 1:** Cases submitted during the 25-week study period.

**Total no of cases submitted**	Excluded cases^*^	**Simple cases**	**Complex cases**
2,161	512	669	980

* *Cases submitted via the Collegium mobile app*.

The median of the differences between the two preparation times was 27 s (IQR 8-146).

Of the 669 cases which were analyzed, 505 cases were submitted with one or more files. In these 505 cases, the median total size of the files uploaded was 1248 kByte (IQR 534-7016). However, there was no association between the size of the files uploaded and the referral preparation time (*r* = 0.11).

The 669 cases were submitted by a total of 129 different referrers. The referrers submitted from one to 75 cases each (median 2).

## Discussion

In a store-and-forward telemedicine system, referral-preparation time is important. A focus-group study of the opinions of potential users of a Canadian store-and-forward system for use in primary care, found that an important barrier to its use was the length of time required for primary-care practitioners to complete the e-referral (6). Designers therefore need to pay attention to the ease with which referral forms can be completed, especially in low-resource settings where there is unlikely to be an existing electronic medical record system, so that referral data will need to be entered manually. We have observed a tendency for specialists to request more and more detailed referral forms as they seek to deal with new requests in a single interaction. However, as referral forms become more complex, the time required to complete them increases. Clearly there is a trade-off between very simple forms which are quick to complete but which require several interactions between referrer and specialist, and very detailed forms which are slow and laborious to complete, but which permit a specialist to provide an answer without further requests for information from the referrer.

The present study shows that the median time taken by users to prepare referrals in the Collegium system was 14.8 min, and the median time spent following login until the referral form was opened was 0.5 min. As far as we are aware, there are no published data concerning the operational use of store and forward telemedicine systems to compare this with. In a teledermatology research study, Nami et al. found that the average time required for writing the letter of referral to the GP, taking photographs and uploading the referral was 14.5 min (7). In an earlier teledermatology research study, Berghout et al. found that the average time from the start of a GP consultation to the submission of the teledermatology referral was 11.5 min (8).

The present analysis was restricted to the simple situation where the user logged in, and – after carrying out other operations such as reading messages concerning other cases – prepared the referral and submitted it. Such cases formed about 40% of all referrals submitted during the study period, i.e., users commonly behaved in a more complex manner, allowing their session to timeout for example, and then logging in again to resume it. Thus, the observation that the median time required to prepare a referral was 14.8 min represents a lower bound on submissions generally.

The present study describes two methods of measuring the time required to prepare and submit a referral. The most accurate method is the direct measurement of referral-preparation time, although this requires the telemedicine system to be able to record the time that the user opens the referral form, and to deal with various complexities in user behavior, such as session timeouts and continuations. Use of the direct method in other, non-Collegium telemedicine networks is therefore likely to require system software changes.

In contrast, the indirect method requires only knowledge of when the user logged in and when the referral was submitted. Thus, indirect measurements of referral-preparation time could in principle be made in most telemedicine networks, since login times and referral submission times are normally recorded as a matter of course. As an example, the indirect referral times were measured in the Collegium system before and after a significant software upgrade had been carried out, on 19 July 2018. This upgrade improved the uploading of user files.

**Figure 1:**
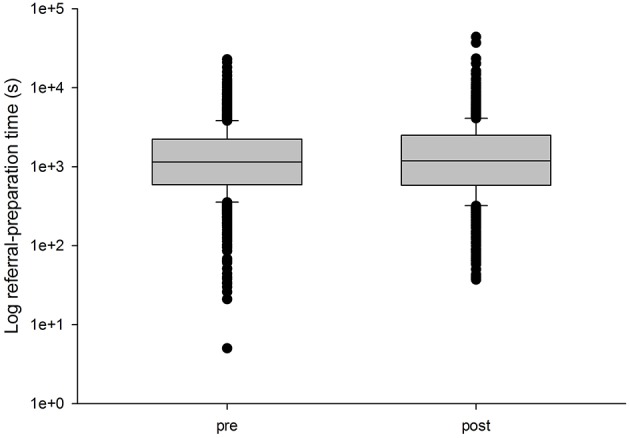
Median referral times in the Collegium system during two 24-week periods immediately before and after a significant system upgrade. The ordinate is shown on a log scale. The lower boundary of the box indicates the 25th percentile, the line within the box marks the median, and the upper boundary of the box indicates the 75th percentile. The whiskers (error bars) above and below the box indicate the 90 and 10th percentiles.

Two 24-week periods were compared, the first ending on 30 June 2018 and the second starting on 1 August 2018. During the first period, 1,402 simple cases were submitted and the median (indirect) referral preparation time was 1,144 s. During the second period, following the system upgrade, 1,323 simple cases were submitted and the median time was 1,182 s, see Figure 1. There was no significant difference between them (Wilcoxon *p* = 0.30). Thus, it seems likely that despite an improvement in file uploading, the effect on the overall time required to submit a referral was too small to be observed.

Part of the process of submitting a referral is that data files containing relevant information (e.g., clinical photographs) must be uploaded. In 75% of the cases studied, one or more files were uploaded – these files ranged in size from 8 kByte to 799.8 Mbyte. Uploading very large files, such as DICOM scan datasets, will take several seconds or even minutes, depending on the speed of the user's connection. However, this upload time does not represent a substantial proportion of the total time required to submit a referral, and in the networks studied there was no correlation between the size of the data files uploaded and the time taken to prepare the referrals. That is, the time spent by the user in composing the text of the referral and typing it in dominated the overall preparation time required. This also suggests that improving the referrer's connectivity, with the aim of reducing file upload times, may not necessarily be a good use of scarce resources.

**Figure 2:**
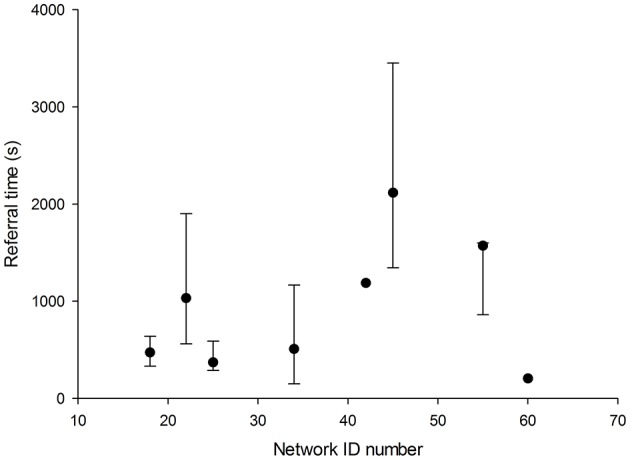
Median referral times in eight Collegium networks (seven clinical and one educational). Three of the clinical networks handled cases of all specialties, and four networks were restricted to specific specialties (radiology, dermatology, and leprosy). The error bars above and below the median indicate the 75 and 25th percentiles.

The main study in the present work concerned 669 simple referrals from a total of eight networks. These networks covered both educational (*n* = 1) and clinical work (*n* = 7). Three of the clinical networks handled cases of all specialties, and four networks were restricted to specific specialties (radiology, dermatology and leprosy). The median referral-preparation times in these networks were broadly similar, see Figure 2. A one-way analysis of variance suggested that the between-network effect was marginally significant (*F* = 2.4, *P* < 0.02). Further work is required, using more extensive datasets.

Given a sufficiently large dataset from which referral-preparation times can be extracted, it would be possible to answer a number of subsidiary questions about how the telemedicine system was being used. For example, it is likely to be of interest to understand how the referral preparation time depends on each of the following factors:

- The source network and the type of cases handled in the network
- The country of the referrer, since certain countries may suffer particularly poor telecommunications which may justify the installation of special-purpose Internet links
- The site of the referral, e.g., specific referral sites may suffer particularly poor telecommunications
- The number and size of any files attached
- The language of the case
- The number of previous referrals by the user, i.e., they may get faster with experience
- The type of referral, e.g., whether about a specific patient or a more general query about a group of patients
- The primary reason for referral, for example whether diagnosis or management advice is being sought
- The referral template used, e.g., general, dermatology, radiology etc.
- The complexity of the case, as measured by the number of specialists being consulted and the number of questions and answers.

With an understanding of these factors, and their effect on the referral-preparation time, suitable measures can be employed to improve the efficiency of referrals.

Referral-preparation time is an important, but little understood, factor in the use of a telemedicine system. Quantitative information about this, and other aspects of the user interface, is important not only in the initial system design, but also in its subsequent development.

## Data Availability

All datasets generated for this study are included in the manuscript.

## Author Contributions

RW and BO'K conceived and designed the study, contributed to writing the manuscript, and read and approved the submitted version.

### Conflict of Interest Statement

RW is a director of Collegium Telemedicus, a not-for-profit organization that provides (free) telemedicine services for humanitarian work in low-resource settings; he receives no salary from Collegium Telemedicus. BO'K was employed by company EndZone.IO Ltd.

## Appendix

### Simple Submission

**Table:**

**Event**	**Time recorded**
User logs in	Time of login, t1
User may read messages about previous cases etc	
User opens referral form	Time of form-opening, t2
User types in referral data (and uploads any associated files)	
User submits referral	Time of submission, t3

Indirect preparation time = t3 - t1
Direct preparation time = t3 - t2

### Excluded (Complex) Scenarios

- Session time-out and resumption of referral. User begins to type in referral data; user interrupted and the session times out. System saves the draft referral automatically. User logs in; user resumes with the referral form; on completion, user submits the referral
- Session time-out and restarting of referral. User begins to type in referral data; user interrupted and the session times out. System saves the draft referral automatically; user logs in, but decides NOT to resume the draft referral, i.e., decides to start again; user opens a new referral form; user submits the referral
- Interruption and resumption of referral. User interrupts preparation and views other pages on the system (e.g., to read messages about another case). System saves the draft referral automatically. User resumes with the referral form; user submits the referral
- Submission of multiple cases. User submits a referral; then submits a second referral (and more) without logging out. Only the *first* referral can be used to calculate the preparation time.

## References

[1] Car J Sheikh A. Email consultations in health care: 1–scope and effectiveness. BMJ. (2004) 329:435–8. 10.1136/bmj.329.7463.435 15321902 PMC514208

[2] Car J Sheikh A. Email consultations in health care: 2–acceptability and safe application. BMJ. (2004) 329:439–42. 10.1136/bmj.329.7463.439 15321903 PMC514210

[3] Wootton R Wu WI Bonnardot L. Nucleating the development of telemedicine to support healthcare workers in resource-limited settings: a new approach. J Telemed Telecare. (2013) 19:411–7. 10.1177/1357633X13506511 24218356

[4] Wootton R Bonnardot L. Experience of supporting telemedicine networks with the Collegium system: first 6 years. Front Public Health. (2019) 7:226. 10.3389/fpubh.2019.00226 31497587 PMC6712066

[5] Huang GK Pawape G Taune M. Telemedicine in resource-limited settings to optimize care for multidrug-resistant tuberculosis. Front Public Health. (2019) 7:222. 10.3389/fpubh.2019.00222 31457000 PMC6700224

[6] Bello AK Molzahn AE Girard LP Osman MA Okpechi IG Glassford J. Patient and provider perspectives on the design and implementation of an electronic consultation system for kidney care delivery in Canada: a focus group study. BMJ Open. (2017) 7:e014784. 10.1136/bmjopen-2016-014784 28255097 PMC5353303

[7] Nami N Massone C Rubegni P Cevenini G Fimiani M Hofmann-Wellenhof R. Concordance and time estimation of store-and-forward mobile teledermatology compared to classical face-to-face consultation. Acta Derm Venereol. (2015) 95:35–9. 10.2340/00015555-1876 24889827

[8] Berghout RM Eminović N de Keizer NF Birnie E. Evaluation of general practitioner's time investment during a store-and-forward teledermatology consultation. Int J Med Informatics. (2007) 76 (Suppl. 3):S384–91. 10.1016/j.ijmedinf.2007.04.004 17532256

